# Nesfatin-1 Neurons in the Ventral Premammillary Nucleus Integrate Metabolic and Reproductive Signals in Male Rats

**DOI:** 10.3390/ijms26020739

**Published:** 2025-01-16

**Authors:** Rege Sugárka Papp, Katalin Könczöl, Klaudia Sípos, Zsuzsanna E. Tóth

**Affiliations:** 1 Human Brain Tissue Bank and Laboratory, Department of Anatomy, Histology and Embryology, Semmelweis University, H1094 Budapest, Hungary; papp.rege@semmelweis.hu; 2 Laboratory of Neuroendocrinology and In Situ Hybridization, Department of Anatomy, Histology and Embryology, Semmelweis University, H1094 Budapest, Hungary; konczol.katalin@semmelweis.hu (K.K.); sipos.klaudia@semmelweis.hu (K.S.)

**Keywords:** ventral premammillary, nesfatin-1, pheromone, hypoglycemia, leptin, *GPR10*

## Abstract

The ability to reproduce depends on metabolic status. In rodents, the ventral premammillary nucleus (PMv) integrates metabolic and reproductive signals. While leptin (adiposity-related) signaling in the PMv is critical for female fertility, male reproductive functions are strongly influenced by glucose homeostasis. The anorexigenic peptide nesfatin-1 is a leptin-independent central regulator of blood glucose. Therefore, its integrative role in male rats can be assumed. To investigate this, we mapped the distribution of nesfatin-1 mRNA- and protein-producing cells in the PMv during postnatal development via in situ hybridization and immunohistochemistry, respectively. Fos-nesfatin-1, double immunostaining was used to determine the combined effect of heterosexual pheromone challenge and insulin-induced hypoglycemia on neuronal activation in adults. We found that ~75% of the pheromone-activated neurons were nesfatin-1 cells. Hypoglycemia reduced pheromone-induced cell activation, particularly in nesfatin-1 neurons. Immuno-electron microscopy revealed innervation of PMv nesfatin-1 neurons by urocortin3-immunoreactive terminals, reportedly originating from the medial amygdala. Nesfatin-1 immunopositive neurons expressed *GPR10* mRNA, a receptor associated with metabolic signaling, but did not respond with accumulation of phosphorylated STAT3 immunopositivity, a marker of leptin receptor signaling, in response to intracerebroventricular leptin treatment. Our results suggest that PMv nesfatin-1 neurons are primarily responsible for integrating reproductive and metabolic signaling in male rats.

## 1. Introduction

One of the most important determinants of reproductive success is the appropriate response to conspecifics, which is highly dependent on sex and metabolic status [1,2]. The integration of sensory and nutritional signals is a complex process that is performed by a tiny hypothalamic nucleus, the ventral premammillary nucleus (PMv), which then regulates reproductive function at the hormonal level via the hypothalamic–pituitary–gonadal (HPG) axis and at the behavioral level through a variety of efferent pathways [3,4,5,6,7]. Consequently, lesions of the PMv have a considerable negative impact on reproductive functions [4,8].

In rodents, cues about conspecifics are transmitted primarily by pheromones. In males, pheromones derived from male competitors elicit aggressive behavior, while pheromones of female origin prompt sexual behavior [5,6]. The PMv cells synthetize a wide range of neurotransmitters [2], which are engaged in the above functions in different ways. For example, dopamine transporter (DAT)−positive neurons in the PMv of male mice respond to male intruders via Fos activation [9], which indicates the active neuronal firing [10], whereas they do not respond in the same way to the presence of females [11]. Indeed, optogenetic activation of DAT neurons results in the manifestation of aggressive behavior, whereas cessation of ongoing attacks is achieved through the silencing of these cells [12]. Conversely, female odors activate (Fos−positivity) numerous cocaine− and amphetamine−regulated transcript (CART) and NADPH-diaphorase-immunoreactive neurons in the PMv of male rats [13,14,15]. Approximately 40% of the CART−positive neurons have been observed to contain the long form of the leptin receptor (*LepR*) mRNA [16], indicating their capacity to respond to the adipose tissue hormone leptin. Leptin production is proportional to adipose tissue mass [17], and leptin, acting both centrally and peripherally, is a known regulator of reproductive functions in both sexes [6,18].

Nevertheless, human studies indicate that a major contributing factor to male reproductive dysfunction is the impaired regulation of glucose homeostasis [19]. Both hypoglycemia and insulin resistance inhibit the HPG axis in men [20,21,22]. Similarly, in male rats, prolonged insulin−induced hypoglycemia results in degeneration of the reproductive organs [20]. This illustrates the importance of the central glucose−sensing mechanisms in the integration of metabolic signaling and reproductive function, which may operate independently of leptin.

Nesfatin−1 is a secreted, anorexigenic fragment of the nucleobindin2 prohormone (NUCB2) acting centrally [23,24] as well as peripherally, e.g., in the gastrointestinal tract [25,26]. Nesfatin−1 exerts its central action through a leptin−independent mechanism [27,28]. Additionally, nesfatin−1 is involved in the control of the reproductive axis in both male and female rats [29,30,31], and its expression is negatively regulated by testosterone in the hypothalamus of males [32]. Furthermore, recent evidence suggests that central nesfatin−1 plays a crucial role in regulating the glucose homeostasis [33,34,35], making it an ideal candidate for involvement in the integration of specific metabolic and reproductive functions.

In accordance with this hypothesis, the objective of the present study was to determine whether nesfatin−1 is produced in the rat PMv and to investigate the function of PMv nesfatin−1 cells. To this end, we examined *NUCB2* mRNA expression and nesfatin−1 immunoreactivity in adult males and during the postnatal development. Furthermore, we characterized the receptor profile of nesfatin−1 neurons, including their sensitivity to leptin, and the afferent innervation of these neurons in adults using in situ hybridization (ISH), immunohistochemistry (IHC), and electron microscopy (EM). We also employed a sexually inexperienced male rat model to study the activation (Fos−positivity) of nesfatin−1 cells in response to female pheromones, as well as the impact of insulin-induced hypoglycemia on this process.

## 2. Results

### 2.1. Nesfatin−1 Producing Neurons in the PMv

**Figure 1:**
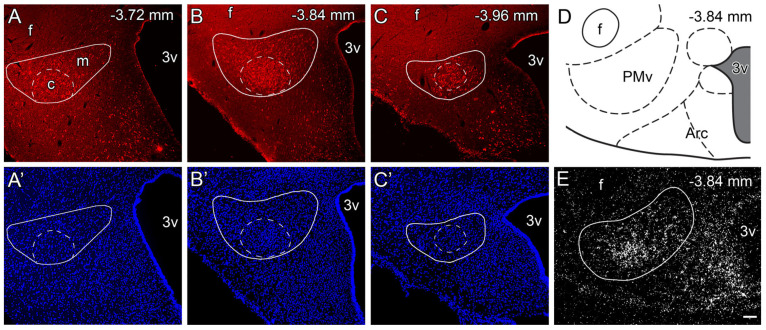
Identification of nesfatin−1−producing neurons in the PMv of male rats. (**A**–**C**) Distribution of nesfatin−1 immunoreactive neurons (red) in the rostrocaudal extent of PMv (outlined with a solid line). Based on the distribution of nesfatin−1−positive cells, a “core” (dashed line) and a “mantle” region are distinguished. (**A’**–**C’**) DAPI−stained corresponding images of (**A**–**C**). (**D**) A schematic drawing of a representative coronal section at the level of the PMv. (**E**) Expression pattern of the *NUCB2* mRNA in the PMv detected via ISH. In the dark-field image, accumulations of silver grains (white, autoradiographic signal) indicate labeled cells. Arc: arcuate nucleus; c: “core” region; f: fornix; m: “mantle” region; PMv: ventral premammillary nucleus; 3v: third ventricle. The rostrocaudal levels from the bregma are indicated in mm. Scale bar: 100 μm for all pictures.

In adult male rats, nesfatin−1 was present throughout the entire rostrocaudal extent of the PMv (Figure 1A–D). Nesfatin−1-immunoreactive neurons were found to be particularly concentrated in the ventral portion of the PMv, designated as the “core”, exhibiting a more dispersed distribution in the remaining portion of the nucleus, henceforth referred to as the “mantle” in this text (Figure 1A–D). A similar pattern of expression was observed with regard to the *NUCB2* mRNA (Figure 1E). The quantitative analysis showed that nesfatin−1−immunopositive neurons made up 25.2 ± 1.8% of the cells in the PMv, based on the DAPI staining (Figure 1A’–C’).

This was about half of the neurons in the PMv, based on the NeuN immunostaining (Table 1). Nesfatin−1 cells were distributed in equal amounts in the “core” and “mantle” regions. However, while the majority of neurons in the “core” exhibited nesfatin−1 positivity, only less than 40% of neurons in the “mantle” were nesfatin−1−positive (Table 1).

**Figure 2:**
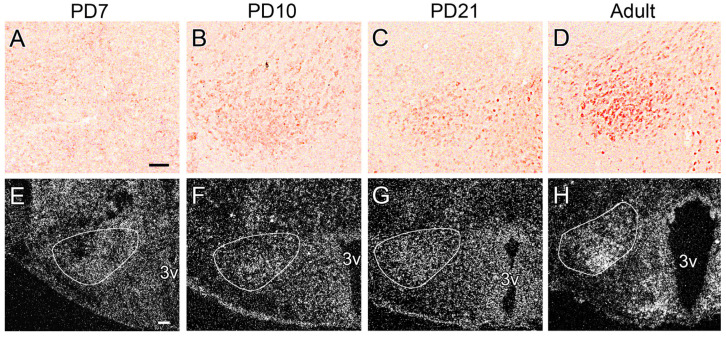
Nesfatin−1 production in the PMv during the postnatal development. (**A**–**D**) Nesfatin−1 immunoreactivity (DAB staining). (**E**–**H**) *NUCB2* mRNA expression. The dark−field images show the result of the ISH. The PMv is outlined. Scale bars: 100 μm.

To gain insight into the *NUCB2* expression and nesfatin−1 immunoreactivity during postnatal development, we conducted IHC and ISH on the brain sections of animals representing different postnatal age groups. The presence of nesfatin−1 immunoreactivity in the PMv was not detectable in neonates at seven days of age (Figure 2A). The initial appearance of nesfatin−1 immunoreactivity was observed at 10 days of age (Figure 2B). Subsequently, nesfatin−1 was consistently expressed throughout development, with the most marked immunoreactivity observed in sexually mature adults (Figure 2C,D). Interestingly, *NUCB2* mRNA expression in neonates was observed to be moderately strong, yet it exhibited no regional distribution (Figure 2E). During the postnatal development period, *NUCB2* expression was observed to be silenced in the surrounding areas but increased in the PMv (Figure 2F–H).

It has been established that the NUCB2 prohormone can be cleaved into two fragments in addition to nesfatin-1. However, the biological functions of these other fragments remain unclear [23]. The moderate overall expression of the *NUCB2* mRNA in the early postnatal period gives rise to the question of whether there are specific developmental factors that influence the cleavage of the NUCB2 prohormone at this particular age.

### 2.2. Functional Characterization of PMv Nesfatin−1 Neurons

**Figure 3:**
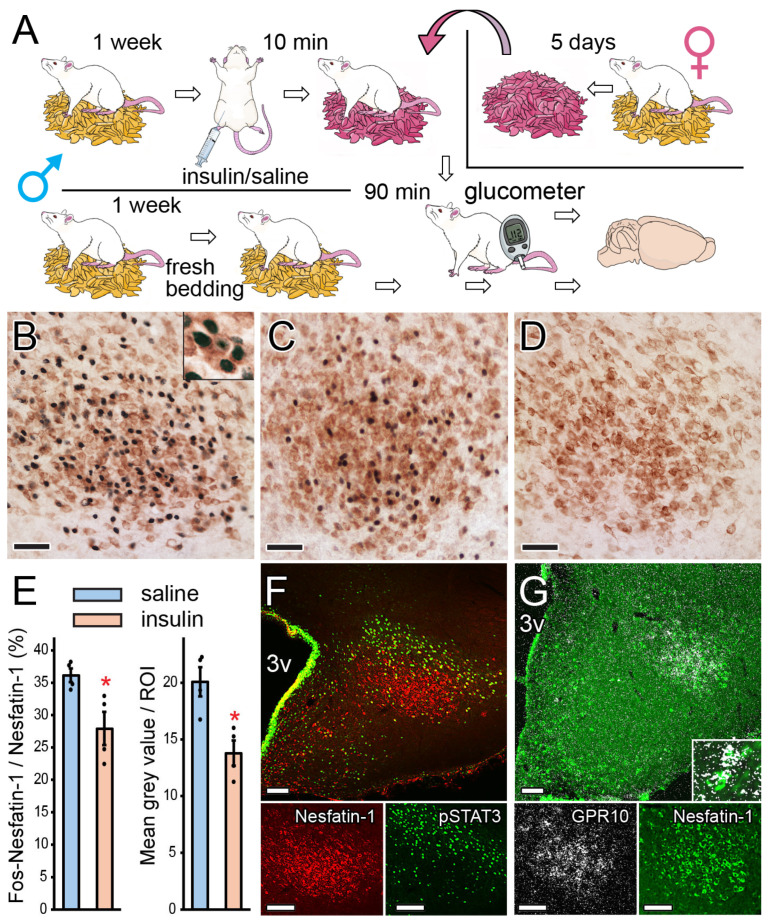
Functional characterization of nesfatin−1 neurons in the PMv. (**A**) Schematic representation of the experimental procedure, in which male rats were challenged with female−soiled bedding and subjected to hypoglycemia along with controls. (**B**–**D**) The result of the nesfatin−1 (brown) and Fos (dark blue) double IHC in pheromone−challenged and saline injected (**B**), pheromone−challenged and insulin treated (**C**), and naive control (**D**) rats. The inset on (**B**) shows a higher magnification of the cells. (**E**) Bar graphs showing the percentage of Fos−activated nesfatin−1 cells relative to the total number of nesfatin−1 neurons in the PMv (left) and the Fos signal intensity in the “core” region (right) of saline− and insulin−treated animals. Left: Mann−Whitney U−test, T = 26, *p* = 0.029; right: Student’s *t*−test, t = 3.70, * *p* = 0.01 (*n* = 4/group). (**F**) The result of the nesfatin−1 (red cytoplasm) and phosphorylated STAT3 (pSTAT3, green nuclei) double fluorescent IHC. The data for the individual channels are shown below. (**G**) The result of the combined *GPR10* receptor ISH (white, silver grains) and nesfatin−1 immunostaining (green cells). The inset shows nesfatin−1 and *GPR10* double−labeled neurons at higher magnification. The pictures at the bottom illustrate the result of the ISH and IHC separately. Scale bars: 50 μm (**B**–**D**) and 100 μm (**F**,**G**).

To explore the function of the nesfatin−1 cell population in the PMv, rats were subjected to heterosexual pheromone challenge combined with insulin−induced hypoglycemia. Briefly, groups of males were kept in separate rooms and isolated from females for one week (Figure 3A). On the day of the experiment, Group 1 received an intraperitoneal (IP) injection of insulin (1.25 IU/kg body weight), while Group 2 received an IP injection of physiological saline solution. Group 3 remained untreated and served as the control group. Ten minutes later, the bedding was changed. The saline− and insulin−injected groups were provided with female−soiled bedding that had been collected from normally cycling female rats for a period of five days, during which time the bedding was not changed. The controls received fresh bedding. The experiment was completed 90 min later, when rats were sacrificed, and their brains were removed for IHC. The blood glucose levels of treated rats were measured from the tail vein just before the end of the experiment (Figure 3A).

Female pheromones strongly activated PMv neurons, including more than one-third of nesfatin−1 cells (Figure 3B,E). Insulin treatment resulted in pronounced hypoglycemia (blood sugar level in mmol/l: saline, 5.68 ± 0.06; insulin, 2.28 ± 0.19; Student’s *t*−test; t = 16.74, *p* < 0.001 (*n* = 4/group)), which caused a significant decrease in the proportion of Fos and nesfatin−1 double−labeled neurons in the nesfatin−1 cell population (Figure 3C,E left). The fresh bedding control group showed minimal cell activation (Figure 3D) affecting 1.04 ± 0.31% of the nesfatin−1−positive cells.

In addition, the intensity of the Fos signal was diminished by hypoglycemia in the “core” region, which predominantly comprised nesfatin−1 cells (Figure 1E, right). Our analysis of the “core” and “mantle” regions separately revealed that pheromone−induced activation of nesfatin−1 cells was adversely affected by hypoglycemia, particularly in the “core” (Table 2). Furthermore, in both the “core” and the “mantle” regions, more than 70% of the pheromone−activated neurons were nesfatin−1 cells (Table 2). The hypoglycemia−induced decrease in cell activation in the “core” was mainly due to a decrease in activation of nesfatin−1 cells, with less effect on other cell types. In contrast, in the “mantle” region, insulin treatment had a comparable impact on nesfatin−1 and other (i.e., nesfatin−1−negative) cells since the proportion of these cells remained unchanged relative to the total number of Fos−activated cells (Table 2).

To further clarify the role of PMv nesfatin−1 cells, we assessed the response of PMv neurons to intracerebroventricular (icv) leptin (2.5 µg/5 µL saline) administration. Leptin signaling in the hypothalamus involves the accumulation of phosphorylated STAT3 (pSTAT3) in the nucleus of cells harboring LepR [36], which can be detected by IHC. Although the leptin−induced pSTAT3 signal was abundant in the PMv, there was a notable spatial separation between the nesfatin−1−positive neurons and leptin−responsive cells, with occasional instances of colocalization (Figure 3F). However, we detected the expression of *GPR10* mRNA, the cognate receptor for the anorexigenic prolactin−releasing peptide (PrRP) [37], in the PMv. This was strongest in the “core” region, where a significant number of nesfatin−1 neurons expressed *GPR10* mRNA (Figure 3G).

### 2.3. Afferent Innervation of PMv Nesfatin−1 Neurons

**Figure 4:**
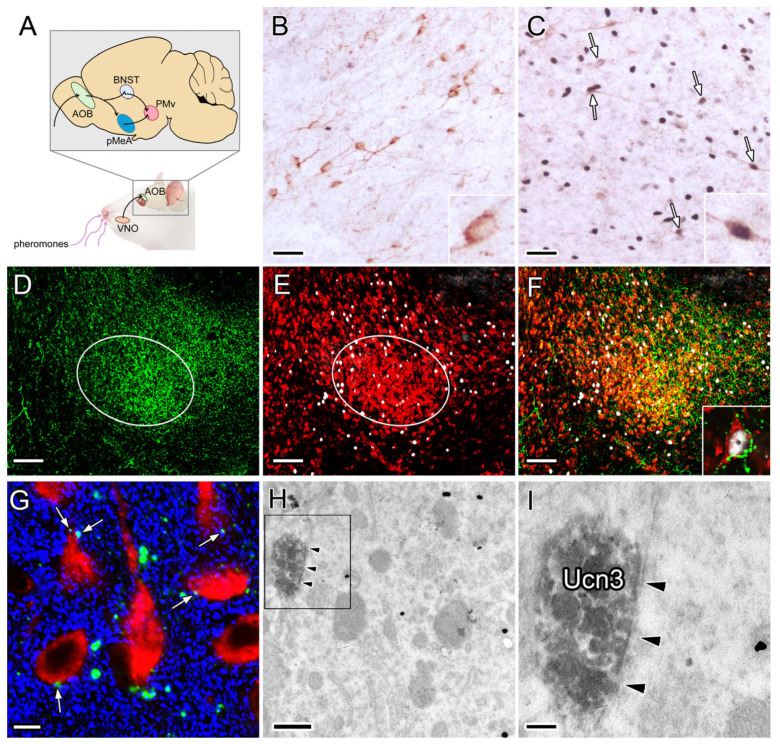
Afferent innervation of PMv nesfatin−1 neurons by Ucn3 axons. (**A**) Schematic of the pathway from the VNO to the PMv. (**B**,**C**) Results of double IHC for Ucn3 (brown) and Fos (dark blue) in the pMeA of naive (**B**) and heterosexual pheromone−challenged (**C**) rats. Arrows point to double−labeled neurons. Insets on (**B**,**C**) show an Ucn3-positive and Fos−negative and an Ucn3 and Fos double−labeled neuron at high magnification, respectively. (**D**–**F**) The result of the triple Ucn3 (green axons), Fos (white cell nuclei), and nesfatin−1 (red cells) immunostaining in the PMv. (**D**) Ucn3 labeling, (**E**) Fos and nesfatin−1 labeling, and (**F**) the overlay picture. The “core” region of the PMv is outlined. The inset on (**F**) shows a Fos and nesfatin−1 double−labeled neuron forming multiple close appositions with Ucn3−containing varicosities. (**G**) Close contacts (arrows) between nesfatin−1 neurons (red) and synaptophysin (blue) −positive Ucn3−containing axon terminals (green). (**H**,**I**) Electron microscopic images showing an axo−somatic synapse between a Ucn3−immunoreactive bouton (DAB labeling) and a nesfatin−1−positive (immunogold−labeled) neuron. Arrowheads indicate a synapse. The framed area in (**H**) is shown in (**I**) at higher magnification. AOB: accessory olfactory bulb; BNST: bed nucleus of the stria terminalis; pMeA: posterior part of the medial amygdala; VNO: vomeronasal organ. Scale bars: 100 μm (**B**–**F**), 5 μm (**G**), 500 nm (**H**), and 200 nm (**I**).

Next, we wanted to explore what type of afferents might transmit heterosexual pheromone signals to PMv nesfatin−1 neurons. It is known that pheromones are sensed by the vomeronasal organ (VNO), which projects to the accessory olfactory bulb (AOB) (Figure 4A).

The AOB, in turn, transmits this information to the PMv primarily via the posterior medial amygdala (pMeA) and the bed nucleus of the stria terminalis (BNST) [38,39,40] (Figure 4A). Odors of the opposite sex activate urocortin 3 (Ucn3) cells in the pMeA, which provide afferents to the PMv [39]. Our experiments confirmed these data; neurons that were double−positive for Fos and Ucn3 were observed in the MeA, intermingled with cells that exhibited a single Fos signal (Figure 4B,C). The PMv was supplied by a dense network of Ucn3 fibers, which were concentrated in the “core” region (Figure 4D–F). The Ucn3−positive axons formed multiple close contacts with pheromone−activated nesfatin−1 immunoreactive cells (Figure 4F). To determine whether the close contacts show synaptic inputs, we first performed triple fluorescent IHC using anti−Ucn3, anti−nesfatin−1 and anti−synaptophysin (a marker for presynaptic vesicles) antibodies. Ucn3−synaptophysin double−labeled axon terminals were identified via confocal microscopy on the cell bodies and dendrites of nesfatin−1−positive neurons (Figure 4G). The existence of synapses between Ucn3−positive axons and nesfatin−1 neurons was further confirmed through EM (Figure 4H,I).

## 3. Discussion

We identified a population of nesfatin−1−positive cells in PMv of male rats. The distribution of these cells delineates two subregions within the PMv, which are designated as the “core” and “mantle” regions in this study.

In accordance with prior findings [13], female−soiled bedding elicited a substantial neuronal activation in the PMv. As was demonstrated, the majority of activated neurons were nesfatin−1−positive cells. In addition, when hypoglycemia was induced by insulin administration, it significantly diminished the neuronal activation elicited by female−soiled bedding, mainly in the nesfatin−1−positive neurons. Consequently, our data strongly imply that the response to heterosexual signals within the PMv is predominantly mediated by nesfatin−1 cells, which are also involved in the integration of reproductive and specific metabolic signals, such as blood glucose and insulin levels, in male rats.

The PMv is a key nucleus in the regulation of pheromone−induced reproductive functions [2,41], and a role for nesfatin−1 in both the neuroendocrine regulation of reproduction [29,30] and the long−term regulation of energy homeostasis [24] has been established. In addition, nesfatin−1 acts as a central regulator of blood glucose level, as chronic central nesfatin−1 treatment improves glucose tolerance and insulin sensitivity [33] and inhibits glucose production in the liver [35] in male rats. In addition, blood glucose is a critical factor in modulating the function of the HPG axis in males. Insulin−induced hypoglycemia disrupts the function of the gonadotropin−releasing hormone (GnRH) pulse generator, the center of the HPG axis, both in rats and men [21,42]. Reduced LH secretion can also be observed in men after two days of fasting [43]. On the other hand, there is a correlation between diabetes and insulin resistance and the occurrence of fertility disorders in humans and rats [44,45], which highlights the significance of maintaining optimal blood glucose levels for the normal male reproductive functions. Thus, the literature data and our results together point to the putative importance of PMv nesfatin−1 cells in coordinating the reproductive function in relation to nutritional status and blood glucose levels. The observation that nesfatin−1 production reaches its peak during adulthood provides further support for this hypothesis.

In accordance with the leptin−independent mechanism of action of nesfatin−1 [27], the nesfatin−1 cells in the PMv did not respond via pSTAT3 accumulation to leptin treatment. Furthermore, leptin−responsive neurons avoided the nesfatin−1-positive “core” region of the nucleus, thereby confirming the existence of a functional distinction between the “core” and the “mantle” regions. In adults, the amount of circulating leptin is proportional to the amount of white adipose tissue and serves as a signal to the brain regarding the amount of stored energy [17]. Accordingly, leptin is regarded as an essential regulator of reproductive functions. Indeed, LepR−deficient male and female mice are infertile [18]. However, while the re−expression of *LepR* specifically in the PMv of LepR−deficient mice induces puberty and restores fertility and GnRH content in young females, this effect is not observed in males [6]. Therefore, although icv leptin administration stimulates LH and prolactin secretion in severely fasted (5 days) adult male rats [46], the primary site mediating this action is likely not the PMv. In fact, leptin signaling in males is thought to play a crucial role during the neonatal period, when a leptin surge is vital for subsequent sexual maturation and fertility. In line with this, leptin levels are markedly diminished in adult males compared to females, which can be attributed to the leptin inhibitory effect of androgens [7].

Although the PMv “core” region exhibited minimal leptin sensitivity, we observed the presence of *GPR10* in the PMv, which was uniquely associated with this region. GPR10 is the principal receptor of the anorexigenic peptide PrRP [37]. Adult GPR10 receptor−deficient rats and mice develop obesity, hyperphagia and decreased glucose tolerance [37,47]. High insulin, leptin and triglyceride levels also characterize the GPR10−deficient mice [37], suggesting a putative role of GPR10 in the PMv in the transmission of signals related to the energy status and metabolism. Notably, GPR10−deficiency affects males more adversely than females [37,48,49]. In comparison to their wild-type littermates, GPR10−deficient males exhibit a significantly greater propensity for obesity with age than females and show decreased energy expenditure with normal food intake prior to the onset of weight gain [37]. Additionally, knock−in mice carrying a human loss of function *GPR10* gene variant, show similar characteristics [48], highlighting the relevance of GPR10 signaling. Unfortunately, no data are available on the reproductive behavior and fertility of GPR10−deficient animals, despite the fact that the GPR10 protein is present in the GnRH cells in the preoptic area [50]. Nevertheless, evidence from both human and rodent studies indicates that GPR10 signaling plays a role in the development of anxiety and mood−related stress disorders [48,51,52], which, in turn, affect reproductive function [53]. Given that nesfatin−1 also functions as a regulator of the stress response [54,55], this suggests a possible complex integrative role for PMv nesfatin−1 cells.

Finally, our study revealed a significant innervation of female odor-responsive nesfatin−1 neurons by Ucn3 fibers. The primary source of Ucn3 cells targeting the PMv is the MeA [56]. Ucn3-positive axons from the MeA have the capacity to transmit a diverse array of information to PMv nesfatin−1 neurons, thereby modulating their function. Ucn3 neurons in the MeA process information related to social behavior and social memory [57,58], and are activated by stress−related signals such as hypoglycemia [59,60,61]. In female mice, the Ucn3 neurons of the posterodorsal (pd) MeA have been demonstrated to mediate the psychosocial stress−induced inhibition of LH release [60]. As previously reported, the majority of Ucn3 fibers innervating PMv are derived from anterodorsal and pdMeA, with the primary source of fibers being the pdMeA [56]. In contrast to the ventral part of the posterior MEA, which is specifically activated by male interaction, the pdMeA is involved in the processing of heterosexual pheromone information [39]. This indicates that the pathways for pheromone-induced reproductive and aggressive behavior are distinct in the MeA. The innervation of nesfatin−1 neurons by Ucn3 fibers, and the finding that those were the nesfatin−1 neurons that were primarily activated by heterosexual odors, suggest that this separation may also exist within the PMv.

In summary, we demonstrated that the PMv contains a large population of nesfatin−1 cells. The distribution patterns of nesfatin−1 neurons, *GPR10* mRNA, leptin−responsive cells, and Ucn3 innervation collectively indicate the existence of two functionally distinct subregions within the PMv: a “core” and a “mantle” region. Furthermore, our data suggest that nesfatin−1 neurons in the PMv are primarily responsible for integrating reproductive and metabolic signaling in male rats. First, female pheromones activated mainly the nesfatin−1 neurons in the PMv, which were leptin−unresponsive, but expressed *GPR10*, a receptor strongly associated with metabolic signaling [62]. Secondly, upon hypoglycemia, a reduction in pheromone−induced neuronal activity was observed in the PMv, and this affected predominantly the activity of nesfatin−1 neurons. It is important to acknowledge that the relatively modest sample size represents one of the limitations of our study. Further clarification of the exact role of nesfatin−1 in the aforementioned processes would be beneficial. One promising approach is to identify the currently unknown receptor for nesfatin−1 and genetically manipulate the levels of PMv nesfatin−1. However, this was beyond the scope of the present work and calls for additional investigations.

## 4. Materials and Methods

### 4.1. Animals and Tissue Handling

The studies were performed on sexually inexperienced male Wistar rats (TOXI-COOP Zrt., Budapest, Hungary) (Table 3). Animals were kept under standard laboratory conditions on a 12:12 h light–dark cycle and fed ad libitum. In all cases, an intramuscular injection of mixture of ketamine (50 mg/kg body weight, Richter Gedeon Nyrt., Budapest, Hungary) and xylazine (4 mg/kg body weight, Medicus Partner Kft., Biatorbágy, Hungary) was used for anesthesia.

The brains were fixed with 4% paraformaldehyde in 0.1 M PBS, pH 7.4, cryoprotected in a 20% sucrose solution, frozen, and stored at −80 °C until use. For EM, 0.08% glutaraldehyde (Merck Life Science Kft., Budapest, Hungary) was added to the fixative solution. For IHC, 50 μm thick free−floating or 20 μm thick slide-mounted serial coronal sections were used. For ISH, fresh−frozen or perfusion−fixed 20 μm thick serial coronal sections were prepared and mounted on positively charged Superfrost UltraPlus slides (Thermo Scientific, Budapest, Hungary).

All experiments were performed in accordance with the National Institutes of Health’s “Principles of Laboratory Animal Care” (NIH Publications No. 85-23, revised 1985), international standards (European Community Council Directive, 86/609/EEC, 1986, 2010), and specific national laws (the Hungarian Governmental Regulations on animal studies 40/2013). The experiments were approved by the National Scientific Ethical Committee on Animal Experimentation, the Ethical Review Board of the Semmelweis University (PE/EA/1563-7/2017, PE/EA/00875-2/2022), and met the guidelines of the Animal Hygiene and Food Control Department, Ministry of Agriculture, Hungary, and the European Communities Council Directive recommendations for the care and use of laboratory animals (2010/63/EU).

### 4.2. Pheromone Challenge

Adult male rats were housed individually in a room isolated from females for one week. To minimize the experimental stress, bedding was changed daily. On the day of the experiment, the animals were divided into groups (*n* = 4/group). Groups 1 and 2 were administered an IP injection of insulin (Humilin R, Lilly Hungária Kft., Budapest, Hungary, 1.25 IU/kg body weight) or physiological saline, respectively. Group 3 remained untreated. Ten min later, Groups 1 and 2 received female−soiled bedding. Group 3 received fresh, unsoiled bedding. Female soiled bedding was collected from normally cycling female rats over a period of five days without bedding change [14]. After 90 min of bedding change, rats were sacrificed under anesthesia. The brains were removed and processed for IHC. Blood glucose levels of treated (Groups 1 and 2) animals were measured from tail vein before anesthesia using a DCont Personal Blood Glucose Meter (77 Elektronika Kft., Budapest, Hungary).

### 4.3. Leptin Treatment

Under deep anesthesia, a polyethylene guide cannula was implanted to the right lateral ventricle (0.8 mm caudal to Bregma, 2 mm lateral from the midline and 4 mm ventral from the skull). The cannula was fixed to the skull with dental cement. After recovery, rats (*n* = 2/group) were fasted overnight and received leptin (2.5 µg/5 µL saline, Phoenix Pharmaceuticals, Inc., Burlingame, CA, USA) or saline via a 26−gauge needle connected to a Hamilton syringe by polyethylene tubing [63]. Animals were sacrificed after 45 min, the brains were removed and processed for IHC.

### 4.4. IHC

A detailed description of each type of IHC is provided in Table 4. Generally, primary antibodies were applied for two days at 4 °C and were diluted in serum containing PBS solutions. Incubations in serum and detection reagents were performed at room temperature for 1 h, and reagents were diluted in PBS unless otherwise indicated. The sections were washed three times in PBS between incubation steps. To prevent non−specific cross−reactions of two primary antibodies raised in rabbit, sections were incubated in anti−rabbit Fab fragment (1:500, 1 h, Jackson ImmunoResearch Europe LTD., Cambrigeshire, UK) or heat treated in a microwave oven before the second IHC [64]. A microwave treatment was also applied to inactivate the horseradish peroxidase (HRP) enzyme−conjugated to secondary antibodies, when required. For IHC before the EM study, sections were frozen four times in liquid nitrogen then treated with 1% sodium borohydride solution for 10 min before processed for IHC

The following primary antibodies were used: rabbit anti−cFos (**#**sc-52, Santa Cruz Biotechnology, Inc., Santa Cruz, CA, USA) [24,65], rabbit anti−Nesfatin-1 (**#**H-003-22, Phoenix) [33,55], rabbit anti−pSTAT3 (Tyr 705) (#9131, Cell Signaling Technology, Inc., Danvers, MA, USA) [66,67], rabbit anti−Ucn3 (PBL #6570, gift from Professor Wylie Vale) [68], mouse anti−NeuN (#MAB377, Merck) [69,70], and mouse anti−synaptophysin (#NCL-L-SYNAP-299, Leica Biosystems-Novocastra, Deer Park, IL, USA) [71,72].

For detections, the following reagents were used: AlexaFluor488 donkey anti−mouse IgG, AlexaFluor568 donkey anti−rabbit IgG, AlexaFluor568 streptavidine, Alexa Fluor568−conjugated tyramide, biotin−conjugated tyramide, DAPI (all from Invitrogen, Budapest, Hungary), polymer−HRP−conjugated goat anti−rabbit IgG, Extravidine−HRP, 3,3′−Diaminobenzidine, nickel sulfate (all from Merck); biotinylated goat anti−rabbit IgG (Vector Laboratories, Newark, NJ, USA), Streptavidine−conjugated Cy5, Fab fragment goat anti−rabbit IgG (Jackson), 0.1% Cold Water Fish Skin Gelatin (CWFS gelatin, Aurion, Wageningen, The Netherlands), Colloidal 0.8 nm gold−conjugated donkey anti−rabbit IgG (Aurion), AURION R−Gent SE−LM kit (Aurion).

### 4.5. EM

Sections were incubated in 1% osmium tetroxide in 0.1 M phosphate buffer (pH 7.4) for 10 min, dehydrated through increasing ethanol concentrations, and flat embedded in Durcupan (Merck) between quick release coated slides (Hobby Time Hold Parting Compound, Electron Microscopy Science, Washington, DC, USA). Serial ultrathin sections were collected on Formvar-coated single−slot grids. Conventional contrast staining was performed using 1% lead citrate in PB for 4 min and 1% uranyl acetate in 70% ethanol for 4 min.

### 4.6. ISH

ISH was performed on coronal sections from adult (*n* = 2) as well as developing (7, 10 and 21 days old) (*n* = 1/age) rats, as described previously [52,55]. Briefly, the rat *nesfatin*−*1* cDNA (246 bp, Invitrogen, GenBank DY314804) and the rat *GPR10* cDNA (189-636 bp, GenBank NM_139193) were cloned into pBC KS+ vector (Addgene, Watertown, MA, USA). The sequence specificity was verified through sequencing and BLAST screening of the rat genome (https://blast.ncbi.nlm.nih.gov/Blast.cgi, (accessed on 8 January 2025)). Antisense riboprobes were generated via in vitro transcription (Maxiscript KIT, Invitrogen) using cDNA templates and [35S]UTP (Per−Form Hungária Kft., Budapest, Hungary). Sections were hybridized with UTP−labeled probes (10^6^ cpm/slide) overnight at 55 °C in a humidity chamber and washed in several steps. The slides labeled with the *GPR10* probe were then processed for nesfatin−1 IHC. At the end of the procedures, all slides were dehydrated, immersed in Kodak NTB emulsion (Carestream Health Inc., Rochester, NY, USA), and stored in the dark at 4 °C for 3 weeks. The autoradiographic signals were developed using Kodak Dektol developer and fixer. Slides labeled with the nesfatin−1 probe were counterstained with Giemsa (all from Merck). All slides were coverslipped with Cytoseal mounting medium (Electron Microscopy Sciences, Hatfield, PA, USA).

### 4.7. Imaging

Microphotographs were captured through an Olympus BX60 microscope (Plan Apochromat 10×/0.25 objective, Olympus, Budapest, Hungary) or using a confocal microscope (Plan Apochromat 20×/0.8 objective, LSM 780, Zeiss, Budaörs, Hungary). EM microphotographs were taken by using a JEOL 1200 EX electron microscope (JEOL GmbH, Freising, Germany). Illustrations were created by using Adobe PhotoshopCS2, Adobe IllustratorCS, and Microsoft Power Point (version: 2410). The schematic drawing of the rat coronal brain section was taken from the Rat Brain Atlas by Paxinos and Watson [73].

### 4.8. Quantitative Analyses

Quantitative measurements were performed using the microphotographs with the help of the ImageJ 1.32j software (Wayne Rasband; NIH, Bethesda, MD, USA) by examiners blinded to the experimental groups. For determining nesfatin−1 cell distribution in the PMv, cells were counted on 5 sections per animal (*n* = 2). The nesfatin−1, NeuN, and DAPI labeled structures were counted in separate channels. To investigate the effect of hypoglycemia on neuronal activation, nesfatin−1 and Fos single− and double−positive cells were counted in three representative sections per rat in the saline− and insulin−treated groups (*n* = 4). The intensity of Fos signal was determined by measuring the mean grey values within a ROI (130 μm^2^) positioned in the PMv “core” in two sections per animal showing single Fos labeling. Background values were measured in parallel and subtracted.

### 4.9. Statistics

Statistical analyses were performed with Sigmastat 3.5 program (Systat Software Inc., Chicago, IL, USA) using the means of the measurements per animal. The normality and equal variance of the data have been tested to ensure that the requisite criteria for statistical analysis have been met. The Student’s *t*−test was used to compare two experimental groups. When the equal variance test failed, the Mann−Whitney U test was employed. A two−way ANOVA followed by Holm–Sidak multiple comparison test was applied to examine the effect of two factors (insulin treatment and area within the PMv) and their interactions. The data are expressed as means ± SEM. Data were considered statistically significant at a *p*−value of 0.05 or lower.

## Figures and Tables

**Table 1:** The proportion of nesfatin−1 immunopositive cells relative to the total number of neurons and distribution of nesfatin−1 cells in the PMv.

Area	Nesfatin-1/NeuN %	Nesfatin-1 %	*n* (Animal/Section)
PMv	52.7 ± 0.3	100	2/5
PMv “core”	84.4 ± 0.1	47.5 ± 1.8	2/5
PMv “mantle”	37.7 ± 2.2	52.5 ± 1.8	2/5

PMv “core” refers to the ventral part of the PMv, where nesfatin−1−positive neurons form a dense cluster, while “mantle” refers to the rest of the PMv.

**Table 2:** Percentage of Fos−nesfatin−1 double−positive cells relative to the total number of nesfatin−1 cells (left) or the total number of Fos−positive neurons (right) in the “core” and the “mantle” regions of the PMv in saline− or insulin−treated, pheromone−challenged animals.

Area	Fos−Nesfatin−1/Total Nesfatin−1 %		Fos−Nesfatin−1/Total Fos %	
	Saline	Insulin	Saline	Insulin
PMv “core”	38.5 ± 1.7	27.7 * ± 1.8	77.0 ± 3.5	69.7 *^#^ ± 1.8
PMv “mantle”	35.4 ± 2.5	28.2 ± 3.2	75.9 ± 1.7	78.3 ± 1.2

Two−way ANOVA, Fos−nesfatin−1/total nesfatin−1: effect of treatment F(1,15) = 14.26, *p* = 0.003. Holm−Sidak multiple comparison test treatment within PMv “core”: p* = 0.008 (*n* = 4/group). Fos−nesfatin−1/total Fos: treatment x area interaction F(1,15) = 4.87, *p* < 0.05. Holm−Sidak multiple comparison test, area within insulin: *p*^#^ = 0.017; treatment within PMv “core”: p* = 0.036, *n* = 4/group.

**Table 3:** The number of rats used in the study.

Purpose	Number
Developmental study (PD7, PD10, PD21) (*n* = 2/age)	N = 6
Pheromone challenge, 3 groups of adults (*n* = 4/group)	N = 12
Leptin treatment, 2 groups of adults (*n* = 2/group)	N = 4
Naive adults for IHC	N = 2
Naive adults for ISH or ISH combined with IHC	N = 2
Naive adult for EM	N = 1
Total:	N = 27

**Table 4:** Description of immunohistochemical reactions.

Subjects	Figure	Pretreatment	1^st^ Antibody	Detection
Naive rats	1	H_2_O_2_ 15 min NDS, Tx	anti-Nesfatin-1 1:3000 anti-NeuN, 1:500	AlexaFluor568-anti-rabbit IgG 1:500 AlexaFluor488-anti-mouse IgG, 1:1000 DAPI
Developing rats	2	H_2_O_2_ 15 min BSA, Tx	anti-Nesfatin-1 1:6000	Biotinylated anti-rabbit IgG 1:1000 EA-HRP 1:3000, DAB
Pheromone challenged rats	3/4	H_2_O_2_ 15 min BSA, Tx	anti-cFos 1:20,000 anti-Nesfatin-1 1:6000 or anti-Ucn3 1:5000	Biotinylated anti-rabbit IgG 1:1000 EA-HRP 1:3000, Ni-DAB Biotinylated anti-rabbit IgG 1:1000 EA-HRP 1:3000, DAB
Leptin treated rats	3	HNSG 20 min BSA, Tx MW	anti-pSTAT3 1:1000 anti-Nesfatin-1 1:6000	Polymer-HRP-conjugated anti-rabbit IgG, 1:4 FITC-tyramide 1:10,000, 10 min Biotinylated anti-rabbit IgG 1:1000 EA-HRP 1:3000, BT 1:20,000, 10 min SA-conjugated AlexaFluor568 1:1000
Naive rats *GPR10* ISH	3	H_2_O_2_ 15 min BSA, Tx	anti-Nesfatin-1 1:3000	Polymer-HRP-conjugated anti-rabbit IgG 1:4 FITC-tyramide, 1:10,000, 10 min
Pheromone challenged saline treated rats	4	H_2_O_2_ 15 min BSA, Tx MW anti-rabbit Fab, 1:500, Na-azide-H_2_O_2_, 20 min	anti-cFos 1:20,000 anti-Nesfatin-1 1:6000 anti-Ucn3 1:5000	Polymer-HRP-conjugated anti-rabbit IgG 1:4 FITC-tyramide 1:10,000, 10 min Polymer-HRP-conjugated anti-rabbit IgG 1:4 AlexaFluor568-tyramide 1:1,000, 10 min Biotinylated anti-rabbit IgG 1:1000 EA-HRP 1:3000, BT 1:20,000 10 min SA-conjugated Cy5 1:1000
Naive rats	4	H_2_O_2_ 15 min BSA MW	anti-Nesfatin-1 1:6000 anti-Ucn3 1:5000 anti-Syn 1:1000	Polymer-HRP-conjugated anti-rabbit IgG 1:4 AlexaFluor568-tyramide 1:1000, 10 min Biotinylated anti-rabbit IgG 1:1000 EA-HRP 1:3,000, FITC-tyramide 1:10,0000, 10 min Cy5-conjugated anti-mouse IgG 1:1000,
Naive rat for EM	4	H_2_O_2_ 15 min BSA anti-rabbit Fab, 1:500 CWFS gelatin, 30 min 1% glutaraldehyde	anti-Ucn3 1:5000 anti-Nesfatin-1 1:3000	Biotinylated anti-rabbit IgG 1:1000 EA-HRP 1:3000, DAB Nanogold-anti-rabbit IgG 1:80, 24 h, 4 °C in CWSF gelatin Silver intensification

BSA: 1% bovine serum albumin (Merck); BT: tyramide-conjugated biotin; CWFS gelatin: 0.1% cold water fish skin gelatin; DAB: 3,3′-diaminobenzidine; EA: extravidine; HNSG: 1% H_2_O_2_, 0.1% NaOH; 0.15% sodium dodecyl sulfate, and 0.3% glycine (all from Merck) diluted in distilled water; H_2_O_2_: 3% hydrogen peroxide solution; MW: microwave treatment in 0.1 M citric acid (pH: 6.0, Merck) for 5 min; Na-azide-H_2_O_2_: 0.01% sodium-azide and 3% hydrogen peroxide solution; NDS: 5% normal donkey serum (Merck); Tx: 0.1% Triton X-100 (Merck); SA: streptavidine; Syn: synaptophysin.

## Data Availability

Data are available within the article. Further inquiries can be directed to the corresponding author.
